# Do Children with Uncomplicated Severe Acute Malnutrition Need Antibiotics? A Systematic Review and Meta-Analysis

**DOI:** 10.1371/journal.pone.0053184

**Published:** 2013-01-09

**Authors:** Gabriel Alcoba, Marko Kerac, Serge Breysse, Cécile Salpeteur, Annick Galetto-Lacour, André Briend, Alain Gervaix

**Affiliations:** 1 Geneva University Hospitals, Child & Adolescent Department, Paediatric Emergency Division, Geneva, Switzerland; 2 University College London, Centre for International Health and Development, London, United Kingdom; 3 Action Contre la Faim, direction scientifique et technique, Paris, France; 4 Department of International Health, University of Tampere, Tampere, Finland; Aga Khan University, Pakistan

## Abstract

**Background:**

Current (1999) World Health Organization guidelines recommend giving routine antibiotics (AB) for all children with severe acute malnutrition (SAM), even if they have uncomplicated disease with no clinically obvious infections. We examined the evidence behind this recommendation.

**Methods and Findings:**

OVID-MEDLINE, EMBASE, COCHRANE, GLOBAL-HEALTH, CINAHL, POPLINE, AFRICA-WIDE-NiPAD, and LILACS were searched for AB efficacy, bacterial resistance, and infection rates in SAM. Following PRISMA guidelines, a systematic review and meta-analysis were performed. Three randomised controlled trials (RCT), five Cochrane reviews, and 37 observational studies were identified. One cohort-study showed no increase in nutritional-cure and mortality in uncomplicated SAM where no AB were used. (p>0.05). However, an unpublished RCT in this setting did show mortality benefits. Another RCT did not show superiority of ceftriaxone over amoxicilllin for these same outcomes, but adressed SAM children with and without complications (p = 0.27). Another RCT showed no difference between amoxicillin and cotrimoxazole efficacies for pneumonia in underweight, but not SAM. Our meta-analysis of 12 pooled susceptibility**-**studies for all types of bacterial isolates, including 2767 stricly SAM children, favoured amoxicillin over cotrimoxazole for susceptibility medians: 42% (IQR 27–55%) vs 22% (IQR 17–23%) and population-weighted-means 52.9% (range 23–57%) vs 35.4% (range 6.7–42%). Susceptibilities to second-line AB were better, above 80%. Prevalence of serious infections in SAM, pooled from 24 studies, ranged from 17% to 35.2%. No study infered any association of infection prevalence with AB regimens in SAM.

**Conclusions:**

The evidence underlying current antibiotic recommendations for uncomplicated SAM is weak. Susceptibility-studies favour amoxicillin over cotrimoxazole. However, given that these antibiotics have side-effects, costs, and risks as well as benefits, their routine use needs urgent testing. With reliable monitoring, we believe that there is sufficient equipoise for placebo controlled RCTs, the only robust way to demonstrate true efficacy.

## Introduction

Severe Acute Malnutrition (SAM) affects nearly 20 million children under five, and contributes to one million child deaths yearly [1], [2]. SAM is an important co-factor of severe infections. Associated with immune-deficiency and respiratory muscle atrophy, SAM triples the risk of mortality from pneumonia, measles, or diarrhoea [1], [3], SAM as defined in the WHO-UNICEF joint statement includes two entities: severe wasting and nutritional oedema [2], [4], [5], [6], [7]. Severe wasting (marasmus), is defined as weight-for-height (WH) below −3 standard deviations (SD or Z-scores), or MUAC (middle upper arm circumference) <115 mm [2], [4], [5], [6], [7]. Nutritional oedema (Kwashiorkor) is defined by bilateral pitting oedema, independently of WH.

The 1999 World Health Organization (WHO) SAM management guidelines focused on inpatients aged 6–59 months, and progressively evolved into Community based Management of Severe Acute Malnutrition (CMAM) [7], acknowledged by WHO, UNICEF and partners in their 2007–2009 statements [2], [6], . Current recommendations for giving routine antibiotics (AB) arose from the time when all children with SAM were treated as inpatients [5].However this recommendation was carried-over into the new outpatient subgroup [2], those with “uncomplicated SAM”, without a specific risk/benefit evaluation.

By definition, uncomplicated SAM children aged 6–59 months present no fever, no sign of infection, nor complicated disease according to IMCI (Integrated Management of Childhood Disease) [9], [10]; they also need to present a sufficiently good appetite to eat a standard amount of Ready-to-Use-Therapeutic-Food (RUTF appetite test); they are treated as low-risk outpatient and comprise about 80% of SAM children [5]. The AB is given for 5–7 days during the first week of the 6–12 weeks outpatient nutritional treatment.

Significant improvements in coverage and mortality were achieved by CMAM [2], [6], [8], [11], but the AB strategy was not adapted. It was impossible to establish benefits attributable to AB: therefore children with uncomplicated SAM still receive AB [6], [7].

Experience from inpatient-based SAM treatment programmes suggested that AB improve survival [12], by treating underlying bacterial complications, possibly undetectable (no fever or tachycardia) in very emaciated bradycardic or hypothermic children [5]. Although AB are part of most guidelines, there has been little analysis of evidence behind the current practice of giving 5–7 days of amoxicillin (AMX) or cotrimoxazole (CTX), in complicated or uncomplicated SAM. These first-line AB are also called “routine” or “systematic” AB, whereas second-line intravenous AB, are given for serious complications. We shall discuss the appropriateness of the first-line AB only.

Regarding the choice of AB, broad-spectrum AB were initially suggested [13],[14], combining standard highly fortified diets oral with AMX, CTX or Metronidazole(MTZ), as first line, or second line injectable Ampicillin (AMP) plus Gentamycin (GEN), plus antimalarials, deworming agents, vitamin A, folate and micronutrients. Treatments showed excellent results on mortality [15], but antibiotic-attributable survival was not studied. AMX is more effective against some bacteria (Streptococcus, Haemophilus, and Enterococci) whereas CTX is more effective against others (Staphylococcus, E.coli, Salmonella, and Shigella), but which bacteria cause more complications in SAM remains unclear.

Reasons to reassess AB in uncomplicated SAM were: high bacterial resistances causing low AB efficacy; low proportions of infections in uncomplicated SAM; added cost and complexity; possible excessive toxicity and allergic reactions. Several million children would be affected by a change of policy.

Our aim was to assess the appropriateness and efficacy of routine first-line antibiotics, AMX and CTX, for children aged 6–59 months with uncomplicated SAM.

## Methods

### Search Strategy

**Figure 1:**
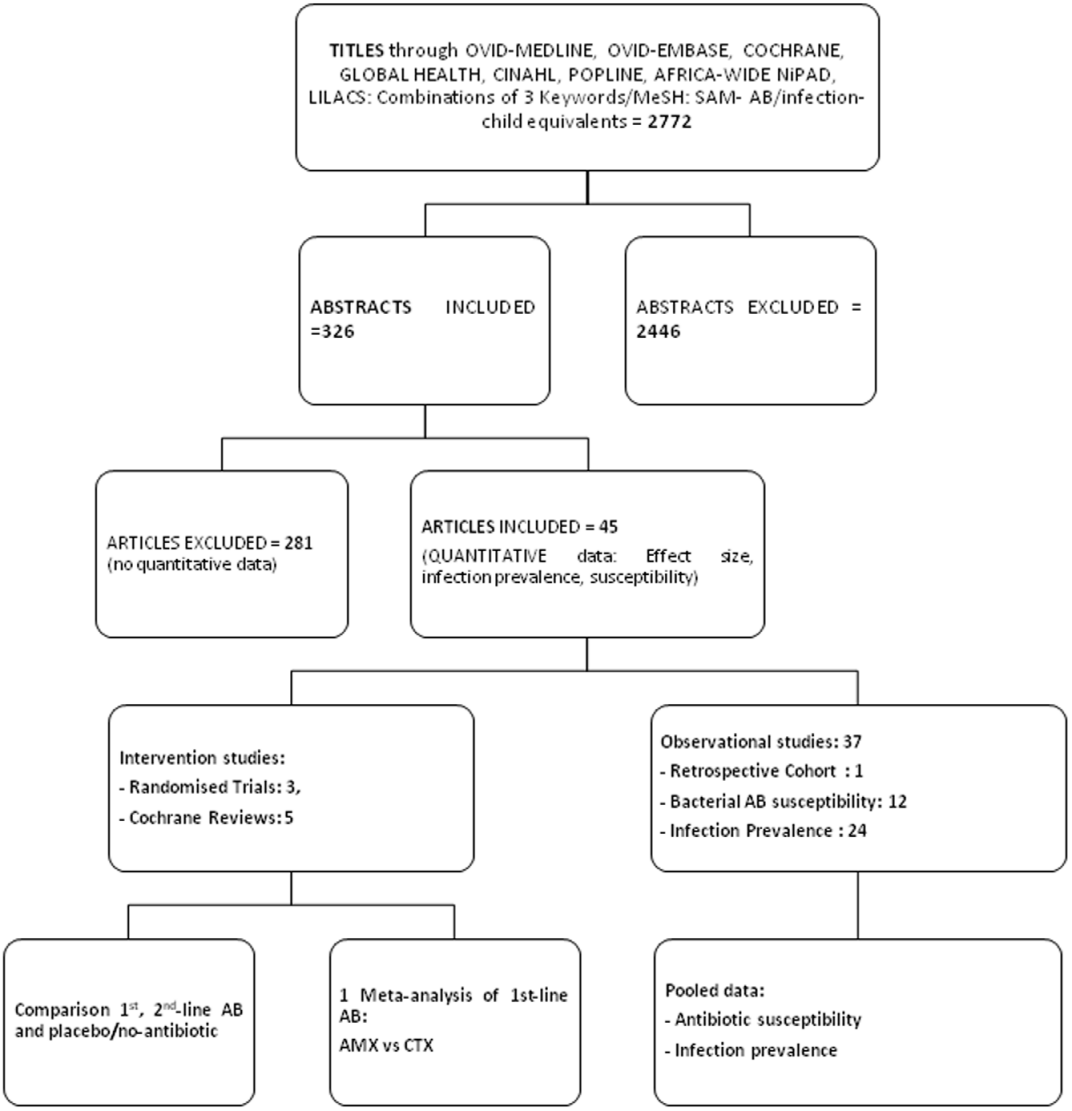
Search results on PRISMA Flowchart (Preferred Reporting Items for Systematic Reviews and Meta-Analyses).

PRISMA guidelines for systematic reviews and meta-analyses were used [16]. We compared international organisations guidelines on antibiotics in SAM. The systematic review addressed the question: “Do children with uncomplicated Severe Acute Malnutrition need antibiotics?”. Sub-questions were: clinical efficacy of AB in SAM; bacterial susceptibilities to AB in SAM; prevalence of infections in SAM according to AB given. Boolean operators and Medical Subject Headings (MESH) were used, through eight databases from January 1960 to August 2011: OVID-MEDLINE, EMBASE, COCHRANE, GLOBAL HEALTH, CINAHL, POPLINE, AFRICA-WIDE-NiPAD, LILACS. We searched articles in English, French, Spanish, Portuguese, and German, and translations from other languages (Chinese, Japanese, and Korean). No geographical limits were applied. We applied inclusion and exclusion criteria by sub-questions and designs. There was no study protocol or registration number. All PRISMA steps were used, including checklist (**File S1**), and search results flowchart (**Figure 1**).

### Selection Criteria

#### Guidelines

Published by international organisations.

#### Studies

Cochrane reviews, randomised controlled trials (RCT), and observational studies on retrospective efficacy (cohort), bacterial AB susceptibilities, bacterial infections in SAM.

#### Participants (inclusions)

Age 6–59 months, plus 0–15 years for possible indirect evidence. Case definitions: SAM children aged 6–59 months with a WH<−3 Z-score and/or bilateral pitting oedema (all references) and/or WH<70% of median and/or MUAC<110 mm (NCHS/WHO 1977 references) and/or MUAC<115 mm (WHO 2006 growth standards) [4]. Marasmus (Severe wasting): WH<−3 Z-score (or <70% of median before 2006), or MUAC<115 mm (<110 mm before 2006). Kwashiorkor: children presenting bilateral pitting oedema, whatever their WH and MUAC, immediately classified as SAM.

*Uncomplicated SAM* = SAM children with successful standard appetite test, without fever, clinical infections, or complications defined by IMCI, treated as outpatient by the lowest health system level, usually a health centre.

*Complicated SAM* = SAM cases without appetite and/or with medical complications (IMCI definitions), treated as inpatients in a hospital setting.

#### Outcomes

AB efficacy was defined as a measure of effect such as odds ratios, risk ratios, or risk reduction% in the following endpoints: case-fatality-rate (CFR), recovery rate, nutritional cure (commonly defined as reaching a weight-for-height within normal range: ≥80% of median or ≥−2 Z-scores), infection incidence (bacteraemia, sepsis, pneumonia, urinary tract infections, meningitis, and diarrhoea), AB susceptibility/resistance.

#### Exclusions

Congenital malformations and chronic diseases, except HIV and TB. No study was excluded due to imprecise calculations of efficacy or measures of effect.

### Search strategy

Search terms: 1) Malnutrition (malnourish*/wast*/undernutrition/undernourished/marasm*/kwashiorkor), 2) Child (pediatric*/paediatric*/infant*/toddler*/newborn), 3) Antibiotic prophylaxis/sensitivity/resistance/antibiotic.mp), amoxicillin (amoxi*/ampicillin/penicillin/clamoxyl) 4) Cotrimoxazole (Bactrim/Cotrim/Septrin/Trimethoprim-Sulphamethoxazole), and combinations 1+2+3, 1+2+4, 1+2+3+4. After a first search finding few quantitative studies, we added search terms: “infection* or bacteraemia* or sepsis or septic*”.

### Data collection and analysis

Results were classified by study type, outcome, relevance (exclusively SAM or not), and age. When possible, results were pooled together. Studies with similar inclusion criteria were eligible for Meta-analysis. Odds ratio (OR) with 95% confidence intervals (95%CI) were the measure of effect for RCT. Susceptibility to AB were summarised as medians with 25–75-interquartile ranges (IQR), and means weighted according to number of subjects (N), and pooled (coefficient proportional to N of each study) into meta-analyses of observational studies [17], using Microsoft Excel™ 2007 and STATA™10.1 (StataCorp, Texas, USA). Forest-plots were not applicable due to absence of comparable measures of effect (OR), so only observational data were meta-analysed.

### GRADE framework

Evidence quality was evaluated through the GRADE framework [18], qualifying studies as good, moderate, low, or very low GRADE with ranks points : RCT initially ranked “good” (4 points), and observational studies as “low” (2), but each study is further assessed: −1 for inconsistency, −1/−2 for (serious) limitations, −1 for bias, −1/−2 for (major) uncertainty. It is increased +1 for strong associations, no plausible confounders, direct evidence (such as adherence to efficacy criteria), +2 for no major validity threats, and +1 for dose-response.

## Results

The review of guidelines is summarized in **File S2**: it showed several differences for the 2 types of first-line AB (AMX and CTX): five different dosages (CTX 4 or 5 mg/kg/d; AMX 50–100 or 70–100 mg/kg/day or three weight-classes) and two different durations (5 or 7 days). Second-line AB are also shown, and highlight guideline diversity [5], [8], [19], [20], [21], [22].

**Figure 1** shows the systematic review search flowchart and numbers of articles. From eight databases, 2772 titles were found, ranging from 15 (LILACs) to 1304 (EMBASE) excluding duplicates. After reading titles, only 326 abstracts were included. Narrowing to quantitative data on AB efficacy, AB susceptibility, and infection prevalence, 45 articles were included: three RCT, five Cochrane reviews, and 37 observational studies.

The data obtained were included in **Table 1** for randomised trials, Cochrane reviews, and a cohort efficacy study, and **Tables 2** **and** **3** for observational studies on bacterial resistance and infection prevalence respectively. Due to heterogeneity in inclusion criteria (SAM, age, and AB) a meta-analysis of intervention studies was impossible. A meta-analysis of observational data was conducted.

**Table 1:** Efficacy of Antibiotics for Severe Acute Malnutrition and paediatric severe infections in efficacy studies (two RCTs and one cohort).

Author, Country, Year	Antibiotics Compared	Design Inclusion (SAM, HIV)	N, Age(m)	Effect Measure	Grade/Relevance
Trehan [23]. Malawi, 2010	**AMX vs No AB**	Retrospective Cohort: 2 centres only; Uncomplicated SAM (all); HIV% = ?	n1:498 n2:1955, 6–59 mths	Recovery worse at 4 wks in AMX group vs no-AMX (40% vs 71%); No difference in recovery at 12 wks (84% vs 86%) p>0.05; Risk of bias due to design and baseline weight+age differences.	2/High
Manary [24], Malawi, 2011	**Cefdinir (CEF) vs AMX vs Placebo**	Randomized double-blinded placebo-controlled: Uncomplicated SAM (all); HIV+ = 188; Tested = 874 (21.5%); Mother HIV = 388	2767, 6–59 mths	Superiority CEF>AMX *>placebo**; Nutritional recovery at 12 wks: 90.9%, 87.7%, 85.1% p* = 0.02 p** = 0.001; Mortality: 4.1%, 4.8%, 7.4% (p = .003); Shorter time to recovery; Risk of BIAS: HIV-NEG not analysed separately.	4/High (But still not in peer-reviewed journal)
Dubray [25], Sudan, 2008	**Ceftriaxone (CRO) vs** (2days-IM) **AMX** (5days-Oral)	RCT non-blinded: Complicated & uncomplicated SAM; HIV% = ?	458, 6–59 mths	No difference in Cured: AMX:70%/CRO:74.6% (p = 0.27); Mortality: AMX3.9%/CRO3.1% (p = 0.67). Cost: 0.2 vs 1.6 Euros (10 kg child); Risk of Bias: complicated SAM included+low power.	3/Medium

**Table 2:** Meta-analysis of observational data on antibiotic (AB) susceptibility (in %) in children with severe acute malnutrition (SAM) or not only SAM (Mixed-NUT).

Author	Country	N	Age	Bacterial antibiotic susceptibility (%)^a^							
			Mths	AMX	CTX	GEN	AGE	CHL	CIP	CRO	COA
**SAM only**											
Babirekere [33]	Uganda	134	6–24	23.3	6.7	33.3	-	60	97	100	-
Bachou (HIV+) [34]	Uganda	30	12–24	53	21	78	-	55	93	84	56
Bachou (HIV−) [34]	Uganda	39	12–24	31	23	81	-	36	93	90	51
Berkley (SAM subgroup) [35]	Kenya	**1182**	7–35	**57**	42	-	87	77	-	94	-
Caksen [36]	Turkey	31	1–30	-	17	100	-	-	82	82	-
Mirabeau [37]	Nigeria	203	<60	-	-	85	-	-	-	61	-
Noorani [38]	Kenya	91	<60	-	-	80	-	-	80	80	-
Rabasa [39]	Nigeria	194	3–60	-	22.7	77	-	-	-	-	22.7
Reed [40]	S. Africa	863	<60	-	-	-	95.8	-	-	-	-
**Median**				***42***	***22***	***80***	***91.4***	***57.5***	***93***	***84***	***51***
***IQR*** †				***27–55***	***17–23***	***77–85***	***87–96***	***46–69***	***82–93***	***80–94***	***23–56***
***Population Weighted*** * ***mean*** ** (Meta-analysis):**				**52.9**	**35.4**	**72.8**	**90.7**	**73.7**	**90.0**	**89.3**	**30.7**
**Mixed-NUT.** ^f^											
Bahwere [41]	Congo	779	1–16	14.3	79.3	100	-	20.7	-	100	-
Bejon(<14 d) [42]	Kenya^d^	690	<14 d	28	71	91	76*^b^*	81	99	95*^c^*	-
Bejon (>14 d) [42]	Kenya^e^	690	>14 d	28	39	73	76*^b^*	62	99	-	-
Berkley (All) [35]	Kenya	**11847**	7–35	**59**	54	-	88	81	-	93	-
Wolff (UTI) [43]	Multiple		<60	12	32	87	-	27	80	67	83
**Median**				**28**	**54**	**89**	**76**	**62**	**99**	**94**	**83**
**IQR** †				**14–28**	**39–71**	**80–96**	**76–88**	**27–81**	**80–99**	**80–97**	-
***Population Weighted*** * ***mean*** ** (Meta-analysis):**				**53.5**	**55.5**	**88.5**	**86.7**	**76.7**	**99.0**	**93.5**	**83.0**

† ***IQR: 25–75 inter-quartile range.***

* Mean susceptibility weighed proportionally (coefficient) to number of patients (N) per study;

a = Cumulated susceptibilities of all isolated bacteria. Abbreviations: AMX = amoxicillin (or ampicillin), CTX = co-trimoxazol, GEN = Gentamicin, AGE = AMX-GEN combination, CHL = chloramphenicol (^b^ 91% susceptible to CHL-GEN); CIP = Ciprofloxacin, CRO = Ceftriaxone (^c^: in this case Cefotaxime instead, similar spectrum); COA = AMX-Clavulanate combination.

d : age: <14 days of life,

e : age>14 days of life.

**Table 3:** Prevalence of HIV, bacteraemia, pneumonia, urinary infections, diarrhoea and mortality, per country, in strictly SAM or not only SAM (Mixed-NUT) children.

Author	Country	N	Age (m)	SAM%	HIV+ %	BACT %	LRTI %	UTI %	DIARR %	CFR %
**SAM only**										
Amadi [44]	Zambia	200	6/24	100	54	17				
Ashraf [45]	Bangladesh	264	6/23	100			35		35	
Bachou1 [34]	Uganda	450	12/24	100	36.7	17.1				
Bachou2 [46]	Uganda	315	12/24	100	39.0	18	68	26	38	
Banapurmath [47]	India	88	<60	100			32	8	27	
Berkowitz [48]	S.Africa	68	<60	100		19*	26	31		18
Berkowitz [49]	Review	1346	<60	100		17.4		22.7		47.4
Christie [50]	Jamaica	50	5/23	100		18	24	24	68	
Friedland	S.Africa	792	<60	100		7.7**				
Jeena [52]	S.Africa		<60	100				38		
Kala [53]	S.Africa	75	<60	100				34.7		
Noorani [38]	Kenya	91	<60	100	43	28.9				
Rabasa [39]	Nigeria	194	3/60	100				11.3		
Reed [40]	S.Africa	323	<60	100		11.8				23
Shimeles [54]	Ethiopia	90	4/60	100		36		37		
Sunguya [55]	Kenya/Tanz.	1121	<24	100		3	18	8	8	
Thame [56]	Jamaica	150	1/31	100		10				
***Pooled***		***5617***				***17.0***	***33.8***	***24.1***	***35.2***	***29.5***
**Mixed-NUT**										
Archibald [57]	Malawi	229	1/156	8	30	15.3				
Bahwere	Congo	779	1/16	30		15.9				
Berkley [35]	Kenya	11847	<60	10		11				12
Blomberg [58]	Tanzania	1828	0/84		16.8	13.9				17
Echave [59]	Senegal	114	2/59	15			11			
Johnson [60]	Nigeria	419	<60	56.8						10.8
Mirabeau [37]	Nigeria	203	<60			36				
Walsh [61]	Malawi	2123	<60***	28	11.2	17.2				37.7
***Pooled***		***17542***				***18.2***	***11***			***19.4***
***Overall Mean***		*23159*				17.4	30.6	24.1	35.2	23.7

Abbreviations: BACT: bacteraemia; LRTI = lower respiratory tract infection (Pneumonia); UTI = urinary tract infection; DIARR. = diarrhoea; CFR = mortality/case-fatality-rate. Oed = oedema. SAM = severe acute malnutrition.

* Bacteraemia cases 6/13 (46%) nosocomial,

** 2.2%;

*** 88% are <5 yrs (including 43%<1 yr+44% 1–5 yr) and 13% 5–15 yrs.

### Randomised trials and other effectiveness studies

Two studies directly addressed our research question: a retrospective cohort-study in Malawi [23]: it compared two therapeutic feeding programs for children 6–59 months with uncomplicated SAM (WH<−3Z), one using AMX for 7 days at 60 mg/kg/day (n = 498), and the other using no AB (n = 1955). It showed worse recovery rates at four weeks in the AMX group (40% vs. 71%), and no evidence of superiority of AMX over the no-antibiotic group at 12 weeks (84% vs. 86%). However these two cohorts were located in two separate districts, and data were collected retrospectively, two major risks of bias. Levels of height and weight for age were lower in the amoxicillin group. Whilst statistical adjustments were made for minor baseline differences in age and anthropometry, unknown confounding or selection bias cannot be excluded, and caution is thus needed interpreting this data.

In contrast, a new RCT in Malawi comparing AMX versus placebo and an oral cephalosporin (Cefdinir) in uncomplicated SAM is published by the FANTA organisation on their website and preliminary non-peer reviewed results suggest superiority of antibiotics over placebo for outcomes such as survival ( ) and nutritional cure () [24].

This highly powered (n = 2767) three-arm RCT shows superiority of Cefdinir over Amoxicillin, and Amoxicillin over Placebo (**Table 1**), showing significantly improved nutritional recovery at 12 wks (90.9%, 87.7%, 85.1% respectively), and mortality (4.1%, 4.8%, 7.4%).

However, it includes 188 children tested HIV-positive, 388 children of HIV-positive mothers, and about 60% of all children had presented fever within the past two weeks. Although children were classified as strictly “uncomplicated “(no fever, no visible complications) it suggests that the infectious risk was high in this HIV endemic area of Malawi. Effect of Antibiotics was not studied in the HIV-negative subgroup. Only one published RCT compared two AB in SAM in a lower HIV area (Sudan): it was randomized but not blinded, nor placebo-controlled, nor outpatient-based, and was underpowered to assess mortality (n = 458) [25]. However, this RCT showed that AMX given 5 days orally was not inferior to intramuscular Ceftriaxone (CRO) given 2 days on SAM recovery-rates.

Three more RCTs and three Cochrane reviews showed less direct evidence for comparison of AB in SAM: they analysed the effectiveness of AB in severe infections (Pneumonia, Measles, and HIV) in populations with high SAM percentages. For paediatric pneumonia, AMX showed equivalent (p = 0.16) [26], or slightly higher recovery rates [OR = 1.33 (1.05–1.67)] compared to CTX [27]. Among children with Measles [28], AB given empirically significantly reduced the incidence of pneumonia, tonsillitis, and purulent otitis media [28], [29]. Finally, among HIV children with a SAM prevalence of 22%, CTX reduced substantially the general mortality and non-*Pneumocystis* pneumonia rates [30], [31].

Among these published RCT and meta-analyses, populations were so heterogeneous for presence of uncomplicated SAM, age, and antibiotics types, that meta-analyses were impossible. **Table 1** shows the three studies that directly evaluate antibiotics in SAM, and it shows three contrasting results: 1) AB not superior to no-AB [23]; 2) CEF superior to AMX and AMX superior to placebo [24]; 3) CEF not superior to AMX [25]. None of these studies provided stratified analyses for HIV+ SAM children.

### Bacterial resistance studies

Data on AB in-vitro susceptibility were analysed to infer possible in-vivo effectiveness. Although these resistance studies are hospital-based, the vast majority of infections are community-acquired e.g. 1590 community- vs 212 hospital-acquired pathogens in one study in Kenya [32]: thus the analysed studies reflected the situation of complicated-SAM children admitted from the community.

Our meta-analysis of observational data from 12 AB resistance studies using bacterial cultures (blood, urine, cerebrospinal fluid) in SAM [33]–[40], and not only SAM children [35], [41], [42], [43], is shown in **Table 2:** among SAM and non-SAM children, our pooled mean susceptibilities were equivalent for AMX and CTX, at 53.5 and 55.5% respectively. Berkley *et al.* showed in a large Kenyan sample (n = 11'847) that AMX and CTX susceptibilities were quite similar (59% vs 54%) [35]. However our meta-analysis of 12 pooled susceptibility**-**studies for all types of bacterial isolates, including 2767 stricly SAM children, favoured amoxicillin over cotrimoxazole for susceptibility medians 42% (IQR 27–55%) vs 22% (IQR 17–23%) and population-weighted-means 52.9% (range 23–57%) vs 35.4% (range 6.7–42%). More resistances to CTX were observed, but there was no association of resistance with mortality, whereas resistance was associated with higher age [35]. CHL and AMX-Clavulanate susceptibilities were respectively 73.7% and 30.7% in SAM. The four antibiotics with lower resistances (susceptibilities >80%) were: GEN, AMX-GEN, CRO, and CIP. These aggregated data, reflected the generally high resistances to AB in a population of mixed, moderate and severely malnourished children, in Sub-Saharan Africa and Turkey.

In summary, cultured bacteria in SAM-children showed very high resistances to first-line AB, such as AMX and CTX.

### Infection and microbial prevalence

The prevalence of clinical infections by syndromes were analysed with regards to the age, country, the HIV prevalence, and the mortality, and subdivided in 2 groups: **Table 3** shows 16 studies of only SAM children [34], [38], [39], [40], [44]–[56], and eight studies of children with not only SAM (Mixed-NUT) [37], [41], [57], [58], [59], [60], [61], where percentage of SAM was included. A total of 21,977 children were included. HIV prevalence was around 40%. Bacteraemia levels ranged from 3% to 30% (mean: 17%). Mortality varied largely from 18% to 47.4% among SAM, and 12% to 37.7% among Mixed-NUT. Pneumonia, diarrhoea, and urinary tract infections (UTI) among SAM-children represented respectively 33.8%, 35.2% and 24.1%.

Other observational studies added valuable data (not in tables): the risk of pneumonia doubled (RR = 2) among children with SAM compared to well-nourished [62]. The risk of bacteriuria was 15.2% among SAM vs 1.8% in controls (p<0.01), and UTI 26.6% vs 5.7% (p<0.05) [63]. Mortality from bacteraemia was 17%, but doubled with SAM, HIV or TB, and was multiplied by five with bacterial resistance [58].

A recent prospective observational study among Kenyan hospitalized SAM children showed the predictive value of a positive urine dipstick (either Leucocyte esterase or Nitrites) on mortality (29% vs. 12%, adjusted HR = 2.5) [64]. It also showed high resistances>50% of Coliforms to Cotrimoxazole and Gentamycin.

We found substantial evidence that Gram-negative bacteria, *Escherichia Coli, non-typhi Salmonella*, and other enterobacteriaceae, represent about 60% (range 58%–77%) and Gram-positive bacteria, mainly *Streptococci* and *Staphylococci*, about 40% of blood-culture isolates [33], [34]. This confirms that children with complicated SAM need effective second-line antibiotics directly, rather than AMX or CTX. However there is no evidence about infection prevalence for uncomplicated SAM outpatients, who (by definition) have excellent appetite tests, no infectious signs, and are alert: they have not undergone bacterial studies for “possibly undetectable” infections.

### Grading evidence

With regards to methodology, relevance and generalisability, following GRADE scoring framework [18], nine studies were considered high grade but not relevant to our question [27], [28], [29], [31]. One RCT [24] available online on the FANTA site, but still not in a peer-reviewed journal qualified as high GRADE: relevant, high-quality in design (ClinicalTrials NCT010000298), high power and low bias for the main outcome, but possibly included some complicated- and HIV-infected SAM children. One RCT [25] was relevant for SAM but medium grade due to unblinded randomisation, and lack of separation of uncomplicated SAM. The 37 observational studies were considered as moderate, low, or very low quality. Six were considered as moderate showing strong associations [33], [34], [35], [41], [46], low risk of confounding and bias, clear multivariate analysis of confounders. The rest of observational studies were low grade. The main limitation was that “uncomplicated SAM” was not addressed separately.

## Discussion

This systematic review found 45 relevant studies, three RCT, five Cochrane reviews, and 37 observational studies. We performed a meta-analysis and pooled-analyses that showed insufficient evidence for current recommendations, and for choosing the appropriate AB, in uncomplicated SAM, if AB are really necessary. Our research is the first one focussing on AB in uncomplicated SAM with a meta-analysis, whereas another very useful recent review [65] has mainly focussed on second-line AB and pharmacokinetics without meta-analysis.

The recommendations to give AB systematically present a much higher level of evidence in case of “complicated SAM” than in case of uncomplicated SAM. Studies showed high-prevalence and mortality from bacterial infections in SAM especially with oedema or HIV. In contrast there is true uncertainty and “equipoise” for the recommendations in uncomplicated SAM.

Interestingly, one Malawian retrospective cohort study [23], where one centre gave AMX routinely and another centre did not, showed some evidence that uncomplicated SAM children can be successfully renourished without AB, with comparable recovery rates. However, caution is needed as biases can be substantial in this retrospective analysis. One large RCT in the same country, Malawi, published on FANTA's website (not yet in a peer-reviewed journal) seems to contradict these first findings, Cefdinir and AMX showing superior recovery and survival rates than placebo. [24]. Unfortunately, in both Malawian studies [23], [24], from high HIV-prevalence rural areas, HIV-negative children were not analysed separately: thus we cannot infer whether “uncomplicated and HIV-negative” SAM children could recover well without AB.

Less relevant RCTs and Cochrane reviews analysed settings where uncomplicated and complicated SAM were not distinguished: oral AMX given 5 days was not inferior to intramuscular Ceftriaxone given 2 days for nutritional cure [25]. It suggested that AMX was still efficacious and that injected Ceftriaxone was not necessary for the majority of SAM children, of which at least 80% are uncomplicated [2]. Between first-line AB, AMX efficacy and resistances did not differ significantly compared to CTX in RCTs and Cochrane reviews [61], but in our meta-analysis we found less resistances for AMX than to CTX in bacterial isolates from strictly SAM children [33], [34], [35].

There is sufficient evidence that bacterial susceptibilities to AMX and CTX are low, but our meta-analysis clearly favoured AMX over CTX for weighted means of AB susceptibility.

Caution is also needed as some studies show good in vivo effectiveness despite high in vitro resistances [30]. Susceptibilities are above 80% for most second-line AB, including CIP. CIP can be given orally and could be considered as first-line in contexts of high resistances or impossible hospital admission. An oral cephalosporin (Cefdinir) is being tested in Malawi in a three-arm RCT against AMX and placebo and could answer our research question [24].

Our pooled analysis of infection-prevalence from 24 studies showed about 17% of children with complicated or uncomplicated SAM presented bacteraemia, and a third presented with pneumonia, urinary infections, or diarrhoea. Although mortality was higher in SAM, the prevalence of bacteraemia was very similar between SAM and non-SAM children: these observational, mainly cross-sectional data cannot infer causality [35]. We would need to test outpatients with uncomplicated SAM to answer our question.

The other limitations of our systematic review were mainly due to the lack of robust studies to address the research question. GRADE evaluations showed insufficient relevance to the research question. Most RCTs had strong internal validity, but lacked generalisability. Observational studies were closer to the research question, but presented heterogeneity and inconsistencies Therefore we minimised the risk of bias of the review as much as possible: “Selection bias” was reduced by subgrouping similar studies. Source bias and database biases were minimized by using a wide range of databases and grey literature sources: LSHTM library, CAPGAN congress, non peer review or unpublished randomized trials [24] books and nutrition guidelines. Publication bias is probably inevitable: we cannot rule out that some research on SAM demonstrated a benefit of amoxicillin on bacterial gut overgrowth and nutritional cure, but the articles do not provide numerical evidence or measures of effect [13].

In conclusion, we found little evidence underpinning current WHO recommendations for AB in uncomplicated SAM [5], except for one RCT which shows superiority of AB, in a high-HIV-prevalence, high-mortality setting . Other RCT and observational studies suggest equipoise for “systematic antibiotics vs no antibiotics”, and low in vitro AMX and CTX susceptibilities, so uncertainty remains: it is important to recognise that there are risks of giving as well as of not giving routine AB. The safety of a no-antibiotics approach depends on staff ability to correctly identify complicated SAM. Meanwhile, in areas with weak health systems, antibiotics could remain a “safety net”. In settings with a stronger health system, not using routine AB saves precious healthcare resources; decreases the risk of unnecessary side-effects and adverse effects, and might also delay the development of bacterial resistance.

With good safety monitoring and carefully selected settings, we believe that there is sufficient equipoise for placebo controlled RCTs, the only robust way to demonstrate true efficacy. Future trials should evaluate antibiotics in uncomplicated SAM in HIV-negative children and in low-prevalence settings.

## Supporting Information

File S1 **PRISMA Checklist (Preferred Reporting Items for Systematic Reviews and Meta-Analyses).**
(DOC)

File S2 **Results of Review of international guidelines chapters for antibiotics in Severe Acute Malnutrition.**
(DOCX)
