# Supplementary material for: Do Children with Uncomplicated Severe Acute Malnutrition Need Antibiotics? A Systematic Review and Meta-Analysis
Source: PLoS One. 2013 Jan 9;8(1):e53184. doi: 10.1371/journal.pone.0053184 (PMC3541371; doi:10.1371/journal.pone.0053184)
Supplement: File S2 — Results of Review of international guidelines chapters for antibiotics in Severe Acute Malnutrition. (DOCX) [file pone.0053184.s002.docx]

**Appendix 1: comparison of guidelines for Uncomplicated and Complicated SAM**

| **Author** | **Year** | **Guideline title** | **Uncomplicated SAM**   - **First-line AB** - Orally | **Complicated SAM**  **Second-line AB**  IV/IM, or orally(=PO) |
| --- | --- | --- | --- | --- |
| **WHO [**[**20**](#_ENREF_20)**]** | 2005 | Hospital care for children | **Cotrimoxazole** x 5 Days  4mg/kg twice daily | **Ampicillin IM** 50mg/kg q6h (Days 1-2), followed by  **Amoxicillin** **PO** (15mg/kg) q8h (Days 3-7)  + **Gentamycin IM** (7.5mg/kg) q24h  (Days 1-7)  If no improvement within 48h add **Chloramphenicol** **IM** (25mg/kg) q8h  (q6h if meningitis suspected) |
| **WHO [**[**19**](#_ENREF_19)**]** | 2003 | Inpatient treatment of SAM children | **Cotrimoxazole** x 5 Days  5mg/kg twice daily |  |
| **WHO [**[**5**](#_ENREF_5)**]** ***** | 1999 | Management of severe malnutrition | **Cotrimoxazole** x 5 Days  5mg/kg twice daily |  |
| **ACF (Action Contre la Faim), Golden MH et al. [**[**21**](#_ENREF_21)**] **** | 2011 | Guidelines for the treatment of SAM | **Amoxicillin** x 7 Days  50-100 mg/kg/day  divided in 2 doses | **add**  **“low-dose” Gentamycin 5mg/kg q24h**  If no improvement or signs of sepsis change to **Co-amoxiclav** plus antifungal (**Fluconazole**) |
| **MSF [**[**22**](#_ENREF_22)**]**  **(Médecins**  **Sans**  **Frontières)** | 2010 | Clinical Guidelines | **Amoxicillin** x 5 Days  (70-100 mg/kg/day)  divided in 2 doses | According to origin:  *Unknown/urinary: 1)* **Ampicillin + Gentamycin**, or 2) **Ceftriaxone + Ciprofloxacin**  *Skin:* **Cloxacillin + Gentamycin**  *Respiratory:* 1) **Ampicillin/Ceftriaxone +/- Gentamycin**, 2) **Coamoxiclav or Ceftriaxone + Ciprofloxacin**  *Digestive/Gynaecological: 1)* **Co-amoxiclav + Gentamycin** or 2) **Ceftriaxone + Gentamycin + Metronidazole** |
| **Valid international [**[**8**](#_ENREF_8)**]** | 2006 | CTC Field Manual | **Amoxicillin** x 7 Days (<10kg: 3x125mg; 10-30kg: 3x250; >30kg: 3x500mg) | **Chloramphenicol PO** (2-5.9 kg: 3x62.5mg; 6-9.9kg: 3x125mg; 10-30kg: 3x250mg) (7 Days) as *outpatient with moderate complications* (e.g. fever not responding). |

*: this AB regimen is used in many national protocols e.g. **Malawi, Kenya**

**: this AB regimen is used in several national protocols e.g. **Ethiopia, Niger.**
